# Multimodal Imaging of a Culotte Bifurcation Procedure in the Left Main Coronary Artery of a Perfusion-Fixed Human Heart: Step-by-Step with Serial Micro-CT Analyses

**DOI:** 10.1007/s12265-023-10369-7

**Published:** 2023-03-20

**Authors:** Thomas F. Valenzuela, Paul A. Iaizzo

**Affiliations:** 1 Visible Heart Laboratories, Department of Surgery, and Institute for Engineering in Medicine University of Minnesota, Minneapolis, MN USA; 2 Medtronic Inc., Santa Rosa, CA USA

**Keywords:** Culotte, Imaging, Bifurcation stenting, Percutaneous coronary intervention, Left main coronary artery, Micro-CT

## Abstract

Percutaneous coronary intervention can be a high-risk procedure that would benefit from optimizing device-tissue interactions between stents and coronary vessels. Using a perfusion-fixed human heart with coronary artery disease, we performed a percutaneous coronary intervention of the left main coronary artery bifurcation. This heart was perfused and multimodal imaging was utilized to view the procedure with direct visualization, fluoroscopy, and optical coherence tomography (OCT). We followed the European Bifurcation Club’s guidelines to perform a single-stent bifurcation before transitioning to a two-stent Culotte technique. After each procedural step, the heart was removed from the perfusion apparatus and transferred to a micro-CT scanner to obtain unique scans. We conducted apposition analyses of the computational 3D models from micro-CT DICOM datasets, and compared them to the results from direct visualization and commercial OCT’s *Apposition Indicator* software. Additional measurements of resulting coronary anatomic expansions were taken to determine the potential roles of each step in improving procedural outcomes.

**Graphical Abstract:**

Micro-CT images show stent deformation during a percutaneous coronary intervention

(provisional to Culotte bifurcation procedure) in an isolated diseased human heart.

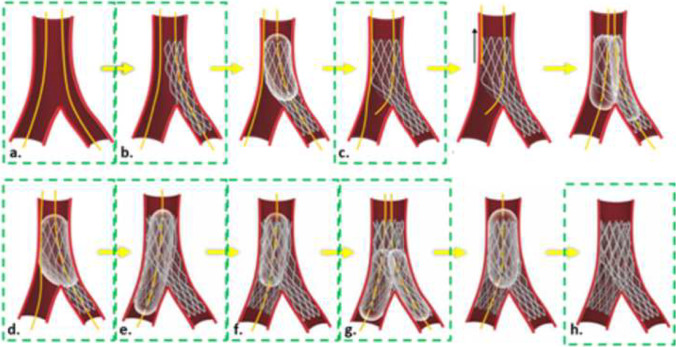

**Supplementary Information:**

The online version contains supplementary material available at 10.1007/s12265-023-10369-7.

## Introduction

Clinical studies and benchtop models are critical in the development, understanding, and optimization of percutaneous coronary interventions (PCIs). Clinically, the use of intracoronary imaging, such as intravascular ultrasound (IVUS) or optical coherence tomography (OCT), has played an important role in identifying features of stent under-expansions and/or mal-appositions [1]. While advancements in intracoronary imaging and its role during PCIs have proven to be useful in predicting clinical outcomes [2, 3], these modalities have their limitations. Additionally, there are groups that utilize silicone models [4, 5] and/or computational simulations [6] of coronary vasculature to study stent behavior, lesion hemodynamics, and artery dimensions. While studies like these have significantly advanced the general understanding and knowledge of bifurcation stenting, they lack the use of true human anatomies and diseased vessel properties.

The Visible Heart Laboratories routinely utilize Visible Heart® methodologies and multimodal imaging to perform PCIs in reanimated swine hearts [7]. While performing PCIs in healthy swine coronaries has its limitations, our laboratory has been able to use perfusion-fixed human hearts [8] (and on rare occasions, reanimated human hearts) to perform bifurcation stenting [9]. These hearts and heart–lung blocs, which are deemed nonviable for transplant, are donated to our laboratory for research through LifeSource (Minneapolis, MN, USA), a nonprofit organ procurement program. The primary goal of the research presented here is to further develop preclinical approaches for assessing and improving our understanding of coronary bifurcation stenting procedures using more clinically relevant specimens. We employed multimodal, OCT, and micro-CT imaging to visualize the step-by-step process of a Culotte bifurcation technique in the left main (LM) coronary of a perfusion-fixed human heart.

## Methods

**Fig. 1:**
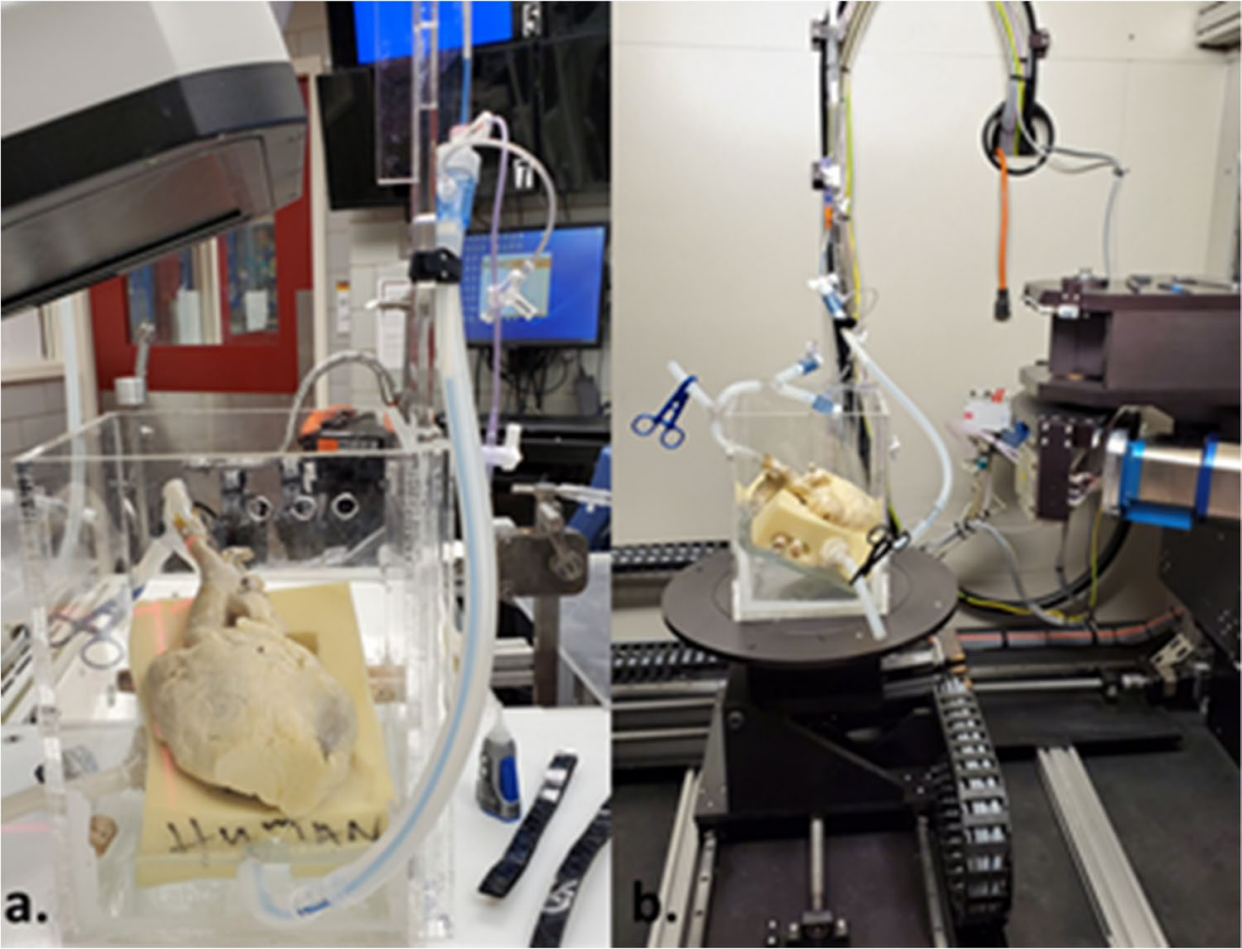
Visible Heart® apparatus. **a** After each key step of the procedure, the tubes were clamped and the descending aorta, guide catheters, and guidewires were tied alongside the acrylic case before being transported to **b** a micro-CT scanner for imaging. The heart was then returned to the laboratory to continue intervention

A donated human heart (68-year-old male), which was perfusion fixed in formalin when received, was selected for this study due to the patient’s history of coronary artery disease. Most of the formalin was removed by rinsing the specimen in water for 24 h. The great vessels were cannulated, and the heart was placed in an acrylic housing where the specimen maintained an anatomical position and orientation during the stepwise PCI procedure (Fig. 1a). Within the acrylic fixture, the heart was attached to a Langendorff perfusion apparatus that continuously perfused the aorta and coronaries with water. Since the specimen was previously fixed in formalin, the use of water as a perfusate, as opposed to a saline (or similar) solution, did not affect the artery’s response during PCI.

Using a combination of direct endoscopic visualization and fluoroscopy, PCI of the LM coronary began by performing a provisional technique, as recommended by the European Bifurcation Club [10–12]. After completion of the initial technique, a secondary stent was added as a means to transition to a two-stent Culotte technique. This entire PCI procedure was performed using Resolute Onyx drug-eluting stents, compliant and non-compliant Euphora balloons, and Cougar XT guidewires (Medtronic, Santa Rosa, CA, USA). All procedural steps were guided and recorded using multimodal imaging with 2.4-mm and 4-mm endoscopic cameras (Olympus, Tokyo, Japan), OEC Elite Fluoroscopy (GE, Boston, MA, USA), and OCT (Abbott Laboratories, Chicago, IL, USA).

**Fig. 2:**
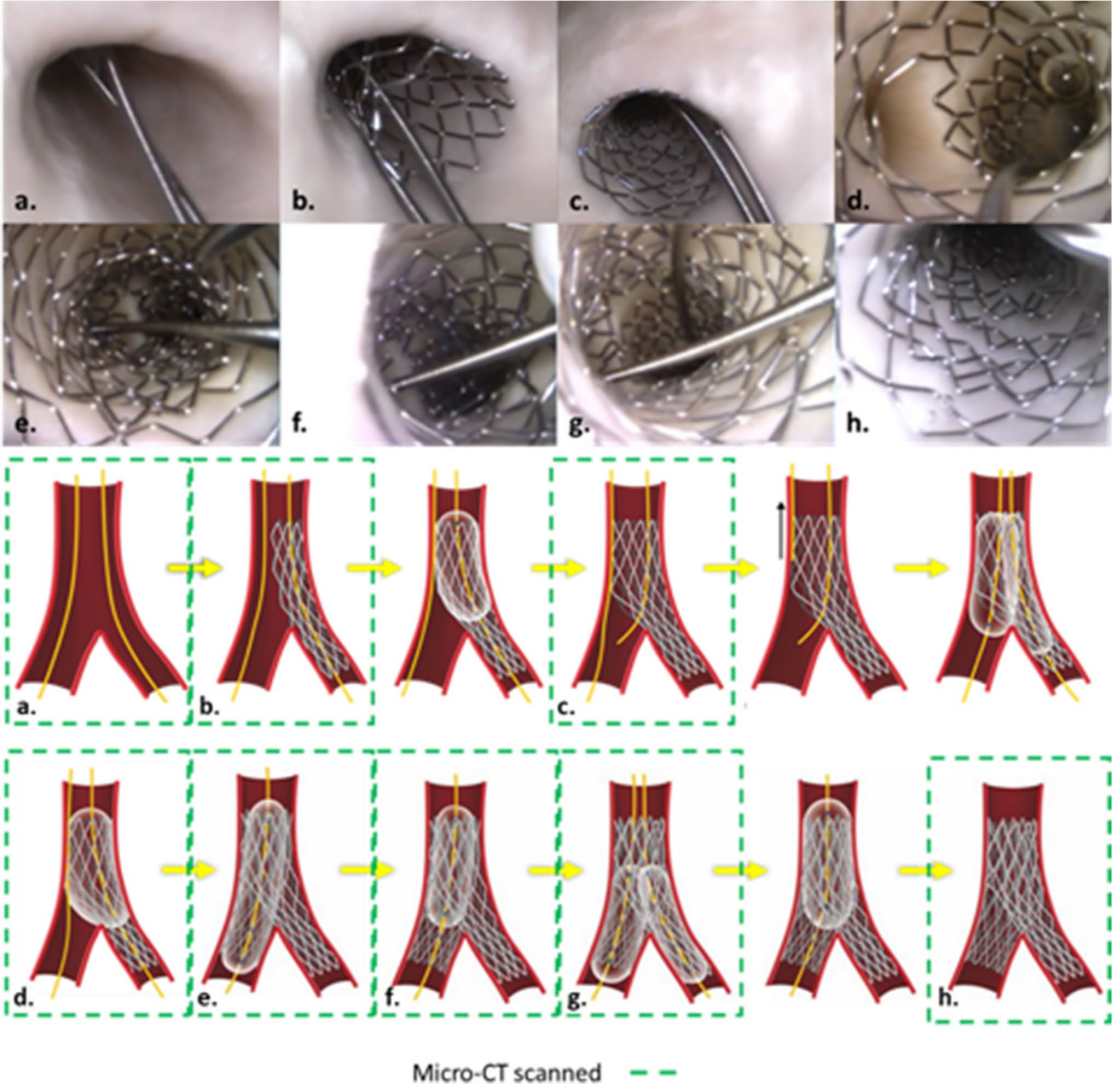
(Top) Direct visualization of the left main ostia was used to guide the operator and capture anatomical changes during percutaneous coronary intervention (PCI). At the eight steps that were micro-CT scanned, endoscopic cameras were used to capture the stent and bifurcation in real time (see also Online Resource 1). (Bottom) Micro-CT scans were taken (a) before PCI, (b) post initial stent deployment, (c) following proximal optimization technique (POT) of the initially placed stent, (d) showing patenting of the side branch using a kissing balloon technique (KBT), (e) after the secondary stent deployment, (f) following POT of the secondary stent, (g) after patenting the main branch using second KBT, and (h) after final POT

Unique to our research, OCT and micro-CT imaging were performed during key steps of the procedure, as highlighted by direct visualization and illustrations in Fig. 2. OCT scans were achieved while the heart was attached to the perfusion apparatus using the automatic pullback sequence with 54-mm scanning length and 5-mm penetrating distance. After initial OCT imaging, perfusion was halted; cannulation tubes were clamped and the heart, along with the acrylic case, was detached from the perfusion system and then carefully transported to an X5000 micro-CT scanner (NSI Imaging, Rogers, MN, USA) located in the University of Minnesota’s Geological Sciences Department. Following each procedural step, the fixed human heart and case were placed in the scanner for imaging (Fig. 1b). Each scan achieved sub-20-micron resolution by utilizing the following parameters: 170-kV tube voltage, 144-µA tube current, 24.5 isowatts, with a scan time of ~ 15 min. After each micro-CT scan, the specimen was returned to the laboratory and reattached to the perfusion apparatus to continue the stepwise PCI until completion. Note that the acrylic casing kept the heart in the same position during transportation to/from the micro-CT scanner, which minimized potential damage to the implanted stent(s) while also being compatible with fluoroscopic imaging and micro-CT scanning.

Upon completion of the PCI procedure and all OCT and micro-CT imaging, we removed the heart from the acrylic case and placed it back in its formalin storage container. Image processing and data analyses were performed following established operating procedures from our laboratory [13]. In short, OCT image datasets were post-processed using the OPTIS Intravascular Imaging System (Abbott Laboratories) to obtain 2D images capturing the reconstructed stent and its resulting appositions using the *Apposition Indicator* function. Micro-CT scans were reconstructed using NSI’s software and exported as 2D images (.tiff) and imported into Mimics, a DICOM analysis software (Materialise, Belgium).

With Mimics, automatic segmentations of the stents using high-density masks, and manually segmented masks of the blood volumes, were created for each step of the PCI process. Once segmented, 3D models were generated from the masks that were then exported into 3-Matic (Materialise) for further study. Each model was composed of tens of thousands of triangular elements (2D shapes with 3 nodes and 3 edges each) that provided spatial information and, when combined, made up the 3D shapes. Uniform remeshing of each model was performed so that each triangular element had a uniform edge length of 50 µm giving each element the same area and thus the same analytical weight, while preserving high model resolutions. The *Part Comparison* tool was then accessed to measure appositions by finding the shortest distances between each element of the stent model to the vessel model, which was generated from the blood volume. Additionally, we performed thresholding to identify and mark the elements with proper apposition (< 200 µm) in green, semi-apposed elements (between 200 and 300 µm) in yellow, and mal-apposed elements (> 300 µm) in red.

Using 3-Matic, we generated a centerline from the 3D blood volume model for each procedural step, including pre-PCI. For each model, a plane normal to the centerline was made in the distal LM, ostial main branch (MB), and ostial side branch (SB) at the closest point to the bifurcation vertex where the centerline first straightened. These planes were used to cut the blood volume into four segments—the LM, LAD, LCX, and polygon of confluence (POC). We then measured the area of each face of the POC by analyzing model properties at each face. The bifurcation angles were calculated using a 3D 3-point method using the bifurcation vertex and the points where the centerline intersected with the face of the MB and SB ostium on the POC. We recorded the bifurcation angles and areas of each face from the series of generated models for comparisons between each procedural step.

After detailed analysis of the 3D models from each PCI step, the resultant*.stl* files were imported into a video game design software Unity (San Francisco, CA, USA) to create virtual reality (VR) scenes with anaglyph capabilities [14, 15]. We rendered each PCI step into its own *scene* where a user can wear a VR headset to interact with the detailed model. By doing so, the user can switch between the various PCI steps and closely observe and compare intricate details of the stent/vessel interactions. Additionally, we also used Unity to create flythrough animations of each PCI step as a resource for lectures, seminars, or in instances when VR capabilities are not available. Finally, the same*.stl* files were used to create 3D prints using uPrint and J750 Digital Anatomy 3D printers (Stratasys, Eden Prairie, MN, USA).

## Results

We performed a provisional stenting technique in the LM coronary artery of a perfused, previously formalin-fixed, human heart. Following the revised MADS-2 classification, this single-stent bifurcation technique was followed with a two-stent Culotte technique and captured via multimodal imaging [16] including (1) 2.4-mm and 4-mm endoscopic cameras, (2) digital fluoroscopy, and (3) OCT (Visible Heart® methodologies). Similar multimodal images can be found in the Atlas of Human Cardiac Anatomy, a free-access educational website (http://www.vhlab.umn.edu/atlas/device-tutorial/stents/index.shtml). Since this stepwise PCI procedure was recorded using small endoscopic cameras, we were able to observe in real time how the stents and associated anatomies were altered after each step (Fig. 2 and Online Resource 1).

**Fig. 3:**
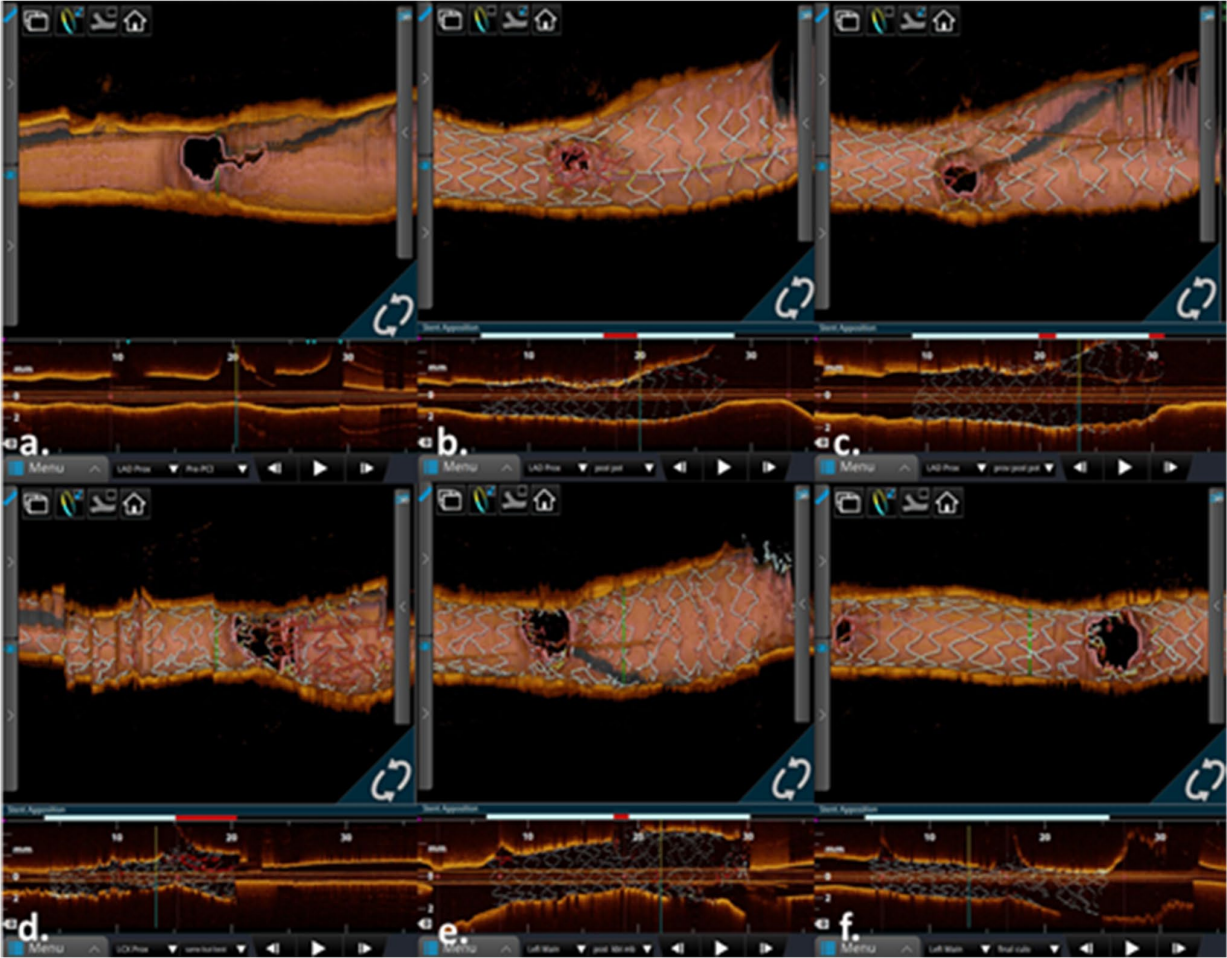
**a** Optical coherence tomography (OCT) scans were taken before percutaneous coronary intervention (PCI). **b** Proximal optimization technique (POT) of the initially placed stent. **c** Patenting the side branch using kissing balloon technique (KBT). **d** After secondary stent deployment. **e** Patenting main branch using a second KBT. **f** And after final POT. Scans were not obtained to capture initial stent deployment and POT of second stent due to the risk associated with advancing OCT catheter

We acquired micro-CT scans at eight key steps of this intervention: (1) before PCI, (2) post initial stent deployment, (3) POT of the initially placed stent, (4) patenting the side branch after a kissing balloon technique (KBT), (5) secondary stent deployment, (6) POT of the secondary stent, (7) patenting the MB using a second KBT, and (8) after the final POT (Fig. 2). In addition, OCT scans and associated reconstructions were performed in all procedural steps, except for steps 2 and 6 due to the operator’s difficulty in advancing the OCT catheter without the possibility of altering or damaging the implanted stents (Fig. 3).

**Fig. 4:**
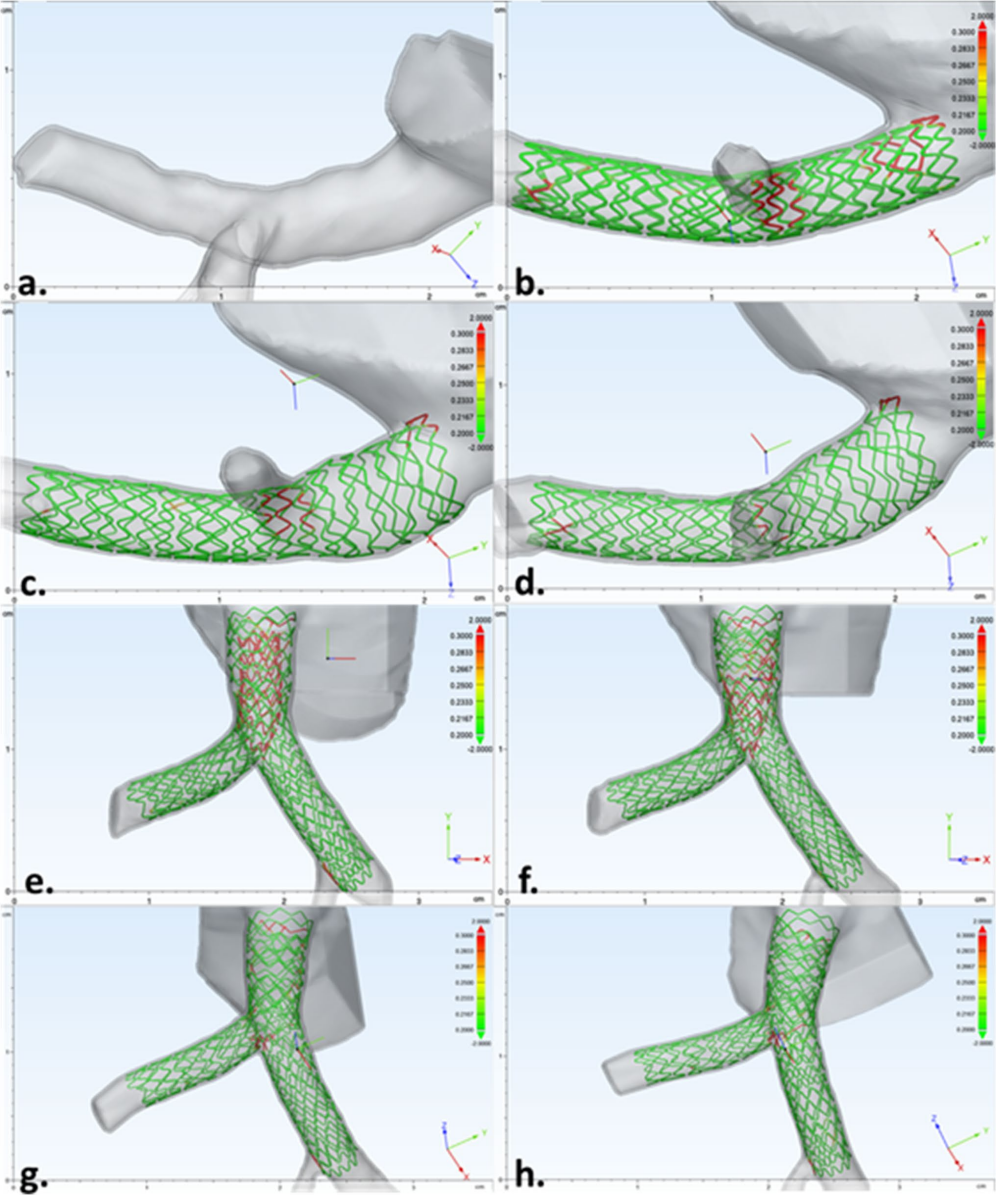
Micro-CT imaging of anatomy before percutaneous coronary intervention (PCI) (**a**) allowed for visualization of diseased anatomy. Stepwise procedure of provisional technique (**b**–**d**) enhanced understanding of how the anatomy, the stent, and its apposition were altered during PCI. After completion of provisional technique, the same stepwise approach was used to perform a Culotte technique (**e**–**h**) from the preexisting provisional. The change in image orientation after the second stent deployment helps to view both stents and appreciate changes in anatomy, stent, and apposition during PCI

**Fig. 5:**
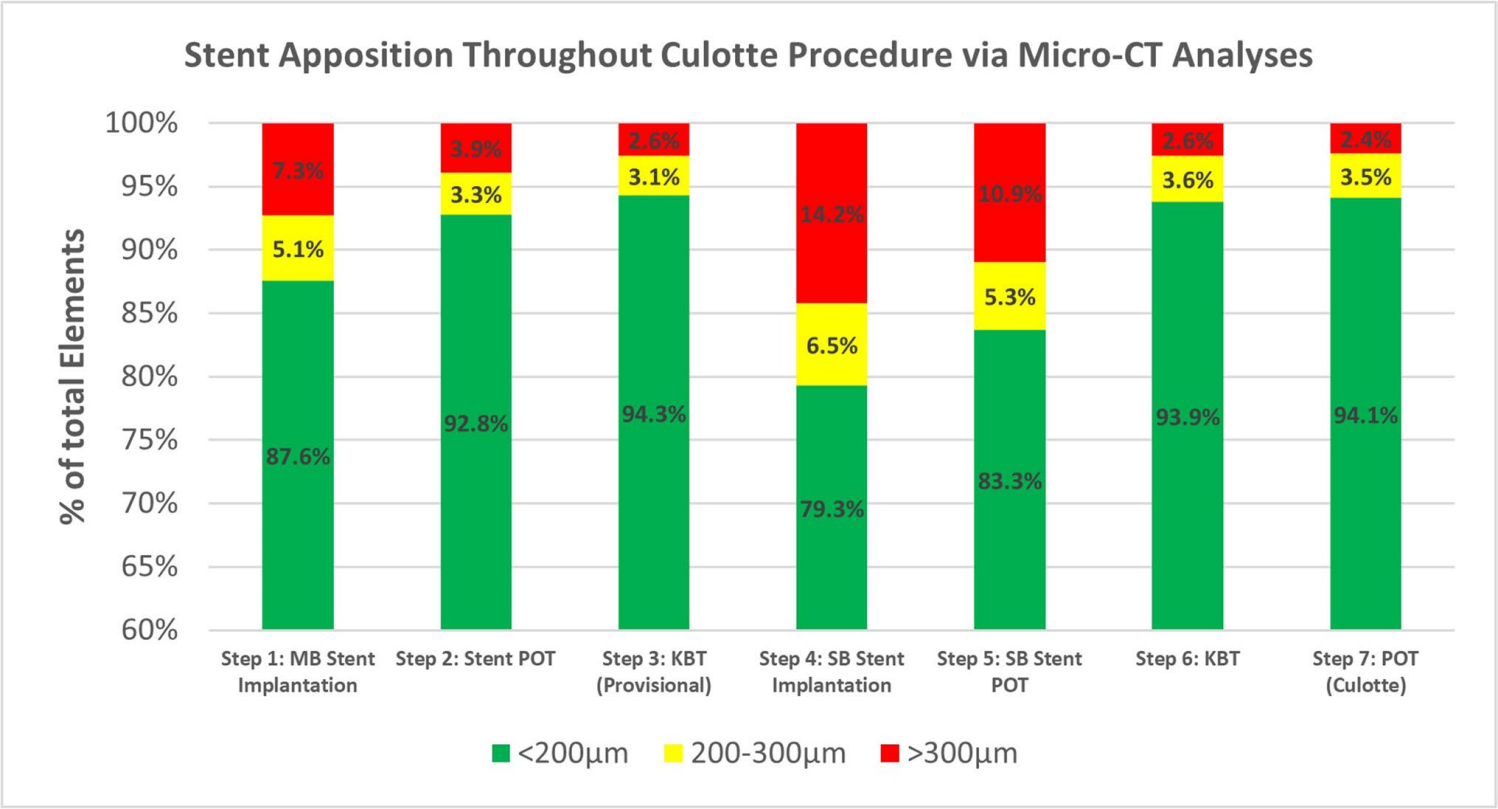
Stent apposition. Distribution of elements according to their apposition from Table 1 was converted into stacked percentage graph, enabling easy representation and comparison of apposition in each procedural step

In this preclinical study, the apposition analyses from each micro-CT scan provided the total number of elements, their location, and resulting apposition (Fig. 4). The data were then categorized into the three classifications from OCT’s Apposition Indicator (< 200 µm, 200–300 µm, and > 300 µm). The resulting models from this micro-CT apposition analyses closely resembled the stent reconstructions and appositions obtained from OCT’s Apposition Indicator. The total number of elements in each category from each step (Table 1) was then displayed as a stacked percentage graph to illustrate how each subsequent step improved overall stent appositions (Fig. 5).

**Table 1:** Apposition analyses of the Culotte stenting procedure

Stent apposition	Step 1	Step 2	Step 3	Step 4	Step 5	Step 6	Step 7
Total elements	100,021	77,210	70,280	130,433	119,908	116,245	129,723
< 200 µm	87,595	71,686	66,299	103,422	100,523	109,132	122,072
200–300 µm	5098	2521	2181	8489	6360	4130	4567
> 300 µm	7328	3003	1800	18,522	13,025	2983	3084

The*.txt* files from *Part Comparison Analyses* were evaluated using a script that returned the total number of elements from the analysis column. The numbers in each column correspond to the apposition of each element (in mm) and were then separated into the different categories from OCT’s *Apposition Indicator*: < 200 µm (proper apposition), 200–300 µm (semi-apposed), and > 300 µm (mal-apposed)

We generated VR scenes and animations from the various PCI step models to create a *Provisional to Culotte* bifurcation module with anaglyph capabilities. Anaglyph scenes allow multiple users to experience 3D aspects of the scenes while navigating along with the proctor, who manages the main VR headset and controls. 3D prints of each PCI step can be made in a variety of sizes, from original scale to 5 × , using various durometers of printing material to accompany our bifurcation module. The progression from performing the PCI in a formalin-fixed human heart to the analysis of stent/stent and stent/tissue interactions to the creation of the mixed reality module is shown in Online Resource 1.

## Discussion

By employing stepwise micro-CT imaging, we obtained a global 3D view of a two-stent PCI bifurcation procedure, thus enabling observation of stent shapes and appositions within the diseased vessels. This unique preclinical benchtop study allows for novel insights and understanding of these types of complex interventional procedures. While similar work has been performed using computational simulations and silicone models, the fact that real human anatomy and tissue were used, without posing a threat to a living patient, is innovative and translates well to current clinical practices.

The PCI described here began with the deployment of a stent, sized 1:1 in the distal MB (LAD), achieving 87.6% proper apposition. Through further manipulations, a provisional stent (minus the final POT) was completed with 94.3% of the stent well apposed, 3.1% semi-apposed, and 2.6% mal-apposed. Because this is a novel way of grading stent apposition (post micro-CT scanning), we determined this to be a clinically satisfactory procedure due to the areas with semi- and mal-apposed elements being located at a smaller bifurcation in the distal MB, the struts of the newly formed metallic carina, and the struts that protruded in the aorto-ostium space. The addition of the secondary stent, sized 1:1 with the distal SB (LCX), initially dropped the total apposition percentage. However, following the guidelines for Culotte stenting [10], we were able to again achieve an apposition percent distribution very similar to the single-stent provisional technique.

Currently, quantitative coronary analysis is a widely accepted method of assessing coronary diameters by performing two 2D angiographies [17]. It has been reported that from these two images, a 3D representation of the coronary anatomy is created, allowing operators to measure diameters, stenoses, and bifurcation angles. However, there is a possibility that the measured diameters and bifurcation angles could vary depending on orientations of the two 2D angiographies. Again, our methods consider all three dimensions and result in the same values despite how the 3D model is orientated and viewed (e.g., when generating centerlines or measuring bifurcation angles).

**Fig. 6:**
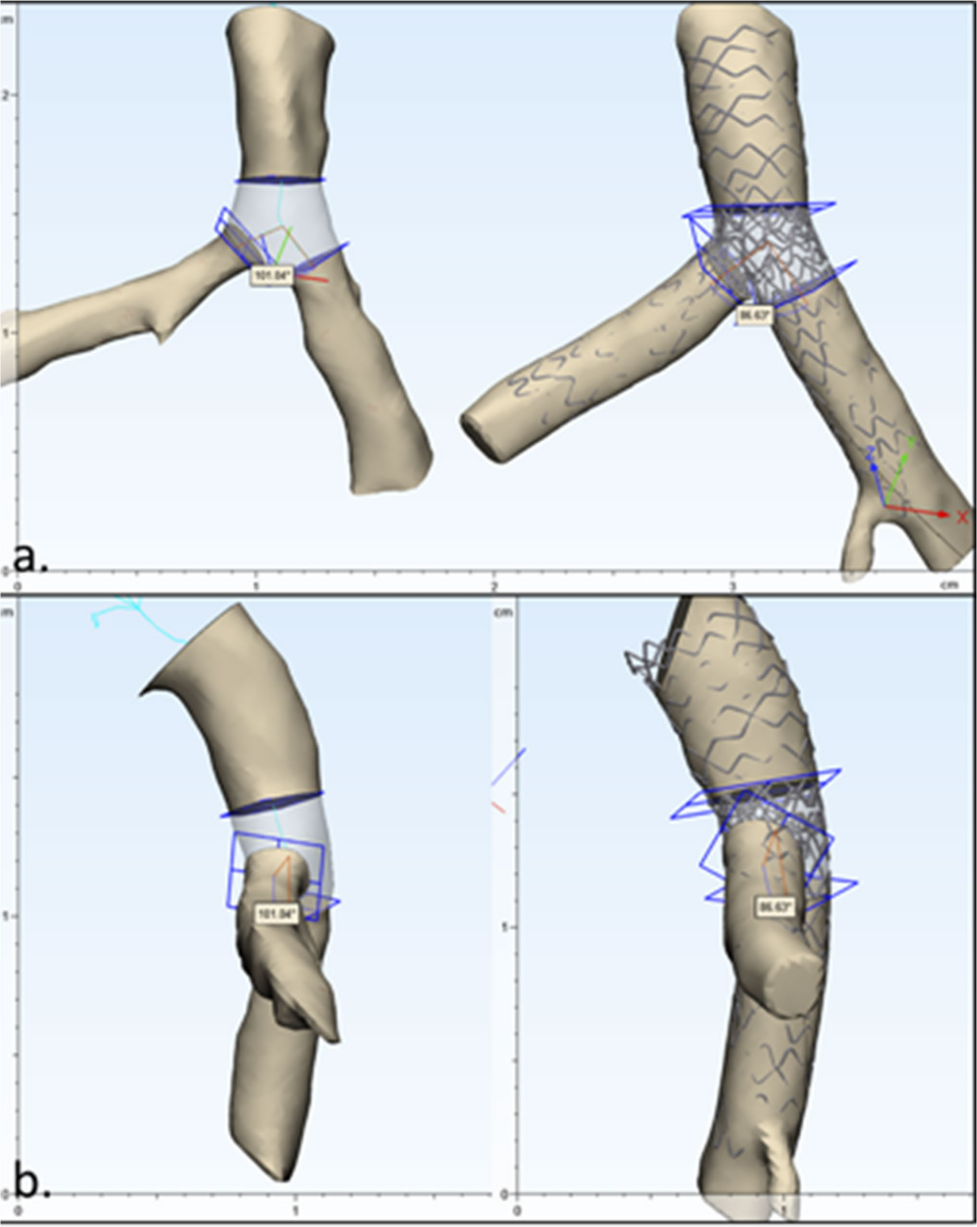
Blood volume and stent 3D models from scans of each procedural step were further analyzed to create consistent polygon of confluence (POC) for each model. The resulting models before and after percutaneous coronary intervention are placed side by side and displayed from **a** posterior and **b** side views. Each model illustrates the centerline, planes along the centerline, bifurcation angle, segments of left main, left anterior descending, and left circumflex artery blood volume (shown in opaque portions), and POC (transparent center region)

The creation of centerlines based on the generated 3D models, and the ability to generate planes normal to the centerlines, allowed us to accurately reproduce the POC within the various bifurcation step models, as demonstrated in Fig. 6. Further analyses of the POC are shown in Table 2; our methods enabled measurement of the areas of each vessel face as well as the resultant bifurcation angles, depicting how each PCI step altered the overall anatomies within this region.

**Table 2:** Analyses of the polygon of confluence

	Distal MV area	Proximal MB area	Proximal SB area	Bifurcation angle
Step 0: Pre-PCI	9.05 mm^2^	6.07 mm^2^	4.66 mm^2^	101.0°
Step 1: MB stent implant	10.33 mm^2^	8.29 mm^2^	3.49 mm^2^	100.1°
Step 2: Stent POT	10.70 mm^2^	8.85 mm^2^	4.63 mm^2^	100.3°
Step 3: KBT 1	11.94 mm^2^	8.49 mm^2^	5.67 mm^2^	89.8°
Step 4: SB stent implant	13.64 mm^2^	8.15 mm^2^	7.00 mm^2^	98.3°
Step 5: SB stent POT	12.93 mm^2^	8.26 mm^2^	7.43 mm^2^	92.2°
Step 6: KBT 2	12.53 mm^2^	9.16 mm^2^	6.82 mm^2^	97.9°
Step 7: Final POT	12.91 mm^2^	9.01 mm^2^	7.08 mm^2^	86.6°

After successfully replicating the polygon of confluence (POC) for each procedural step, we measured area of the faces corresponding with the distal main vessel (MV), proximal side branch (SB), and proximal main branch (MB), as well as the bifurcation angle between the faces using the generated centerline. This allowed for comparison between different models to better understand how the procedural steps affect the area of blood volume and bifurcation angle

*KBT*, kissing balloon technique; *PCI*, percutaneous coronary intervention; POT, proximal optimization technique

While multimodal imaging was considered useful in providing direct visualization of the procedural steps that could help us interpret the accuracy of OCT and micro-CT images, it may be considered a potential study limitation. For example, the use of direct visualization during PCI may have resulted in better outcomes than what is normally observed clinically, i.e., showing higher percentages of well apposed struts. Therefore, future studies could include the replication of this study without the guidance of direct visualization. We believe that a more important direction would be to perform these stepwise PCIs using a variety of different bifurcation techniques and/or various procedural steps, to assess their impact on apposition. Such studies are ongoing in our laboratory, specifically observing how these procedural steps affect not only associated anatomies, but also flow patterns.

We believe that the procedural videos gathered using multimodal imaging and subsequent flythrough animations provide novel imaging and insights for this bifurcation procedure. These have been uploaded on the Atlas of Human Cardiac Anatomy website as an educational resource for interventional cardiologists, students, and medical device designers. In addition, our mixed reality educational platform, composed of both VR and 3D prints, has been shown to improve learning capabilities and retention when compared to traditional single modality teaching methods [15].

## Conclusion

In this study, we uniquely performed a stepwise Culotte LM bifurcation stenting procedure in a perfusion-fixed human heart presenting with coronary artery disease. We utilized novel apposition analyses to investigate the associated anatomical changes and stent appositions at key steps in the intervention. While the present study highlights results based on currently accepted procedural recommendations for a two-stent Culotte technique, future studies could focus on altered or new techniques. More importantly, because of our laboratory’s unique platform, two-stent bifurcations can be performed to show the complications and/or consequences of improper alignment/inflations, cell crossings, etc. The intraprocedural images, computational models, and analyses presented here are original and offer novel educational insights that can be utilized by students, residents, clinicians, and those developing PCI technologies. 

## Supplementary Information

Below is the link to the electronic supplementary material.

Supplementary file1 (MP4 145306 KB)

## Data Availability

All data is presented here and the procedural videos and 3D models can be found on the Atlas of Human Cardiac Anatomy (http://www.vhlab.umn.edu/atlas/device-tutorial/stents/index.shtml).

## Abbreviations

KBT: Kissing balloon technique
LAD: Left anterior descending
LCX: Left circumflex artery
LM: Left main
MB: Main branch
OCT: Optical coherence tomography
PCI: Percutaneous coronary intervention
POC: Polygon of confluence
POT: Proximal optimization technique
SB: Side branch
VR: Virtual reality

## Declarations

### Animal Studies

No animal studies were carried out by the authors for this article.
